# Improving Response Inhibition in Parkinson’s Disease with Atomoxetine

**DOI:** 10.1016/j.biopsych.2014.01.024

**Published:** 2015-04-15

**Authors:** Zheng Ye, Ellemarije Altena, Cristina Nombela, Charlotte R. Housden, Helen Maxwell, Timothy Rittman, Chelan Huddleston, Charlotte L. Rae, Ralf Regenthal, Barbara J. Sahakian, Roger A. Barker, Trevor W. Robbins, James B. Rowe

**Affiliations:** a Department of Clinical Neurosciences, University of Cambridge, Cambridge, United Kingdom; b Department of Experimental Psychology, University of Cambridge, Cambridge, United Kingdom; c Cambridge Cognition Ltd, University of Cambridge, Cambridge, United Kingdom; d Behavioural and Clinical Neuroscience Institute , University of Cambridge, Cambridge, United Kingdom; e Medical Research Council Cognition and Brain Sciences Unit, Cambridge, United Kingdom; f Division of Clinical Pharmacology, Rudolf-Boehm-Institute of Pharmacology and Toxicology, University of Leipzig, Leipzig, Germany; g Behavioural and Clinical Neuroscience Institute, Cambridge, United Kingdom

**Keywords:** Atomoxetine, Impulsivity, Noradrenaline, Parkinson’s disease, Response inhibition, SSRT

## Abstract

**Background:**

Dopaminergic drugs remain the mainstay of Parkinson’s disease therapy but often fail to improve cognitive problems such as impulsivity. This may be due to the loss of other neurotransmitters, including noradrenaline, which is linked to impulsivity and response inhibition. We therefore examined the effect of the selective noradrenaline reuptake inhibitor atomoxetine on response inhibition in a stop-signal paradigm.

**Methods:**

This pharmacological functional magnetic resonance imaging study used a double-blinded randomized crossover design with low-frequency inhibition trials distributed among frequent Go trials. Twenty-one patients received 40 mg atomoxetine or placebo. Control subjects were tested on no-drug. The effects of disease and drug on behavioral performance, regional brain activity, and functional connectivity were analyzed using general linear models. Anatomical connectivity was examined using diffusion-weighted imaging.

**Results:**

Patients with Parkinson’s disease had longer stop-signal reaction times, less stop-related activation in the right inferior frontal gyrus (RIFG), and weaker functional connectivity between the RIFG and striatum compared with control subjects. Atomoxetine enhanced stop-related RIFG activation in proportion to disease severity. Although there was no overall behavioral benefit from atomoxetine, analyses of individual differences revealed that enhanced response inhibition by atomoxetine was associated with increased RIFG activation and functional frontostriatal connectivity. Improved performance was more likely in patients with higher structural frontostriatal connectivity.

**Conclusions:**

This study suggests that enhanced prefrontal cortical activation and frontostriatal connectivity by atomoxetine may improve response inhibition in Parkinson’s disease. These results point the way to new stratified clinical trials of atomoxetine to treat impulsivity in selected patients with Parkinson’s disease.

Dopaminergic drugs remain the mainstay of Parkinson’s disease (PD) therapy but often fail to improve cognitive and behavioral problems such as impulsivity and poor executive control (1, 2, 3). Their limited effect may be because they do not address the loss of other monoaminergic projections to the forebrain, including noradrenaline (4, 5, 6), or changes in the frontostriatal connections that support inhibition and other executive functions (7). Noradrenergic agents have been suggested as a treatment for impulsivity and executive dysfunction (8, 9, 10, 11). This study focuses on one important facet of the multidimensional construct of impulsivity, the impairment in response inhibition.

We proposed that noradrenaline plays a crucial role in regulating the neurocognitive systems for response inhibition in the context of PD, based on preclinical evidence from animal and human studies. We distinguished the prevention of prepotent action (restraint) from stopping an initiated action (cancellation). These two forms of response inhibition are typically studied with the NoGo paradigm and stop-signal reaction time (SSRT) task, respectively. In rats and humans, the selective noradrenaline reuptake inhibitor atomoxetine improves stop-signal performance (12, 13, 14, 15, 16), reduces premature decisions (17, 18), and increases right inferior frontal gyrus (RIFG) activation (19). In contrast, a similar dose of atomoxetine had no effect (or even negative impact) on NoGo inhibition in healthy adults (20, 21), which supports preclinical evidence of a neuropharmacologic distinction between restraint and cancellation type inhibition (22, 23). Evidence for a noradrenergic benefit on stop-signal performance also comes from human and animal studies of methylphenidate, a reuptake inhibitor of both noradrenaline and dopamine (24, 25, 26, 27, 28). The beneficial effect of methylphenidate on SSRT might, in principle, be due to either dopaminergic or noradrenergic mechanisms alone, but noradrenergic and dopaminergic systems may also act synergistically (27), which is of particular relevance to the possible use of noradrenergic drugs for adjunctive treatment of PD.

Using pharmacological functional magnetic resonance imaging (fMRI), we examined the effect of atomoxetine (40 mg, versus placebo) on response inhibition in PD. A favorable impact of atomoxetine on SSRT was identified in a preliminary double-blinded randomized crossover behavioral study (29). This study went further, using fMRI to reveal the mechanisms of atomoxetine’s effect and identifying potential predictors of treatment benefit. We studied the impact of atomoxetine on SSRT and NoGo inhibition, using a task incorporating both NoGo and stop-signal trials.

We predicted 1) that action restraint (NoGo) and cancellation (SSRT) are both impaired in PD (30, 31); 2) that atomoxetine selectively enhances activations of the RIFG on successful stop-signal trials; and 3) that the behavioral effects of atomoxetine would depend not only on the stop-signal RIFG activation but also on the RIFG-striatum connectivity that links the inhibition with motor systems.

## Methods and Materials

### Participants

Twenty-one patients with PD (United Kingdom PD Society Brain Bank Clinical Diagnostic Criteria) (32, 33) were recruited via the Cambridge University PD Research Clinic. Inclusion criteria were 1) Hoehn and Yahr Scale 1.5 to 3; 2) age 45 to 80; 3) English speaking; 4) right handed; and 5) nondemented (clinical impression and Mini Mental State Examination >26/30). Patients were excluded by 1) clinically significant current depression; and 2) contraindications to magnetic resonance imaging (MRI) or atomoxetine. None of our patients had impulse control disorders. All of them were tested on regular PD medication, including levodopa (*n* = 19), nonergot dopamine agonists (*n* = 17; 8 pramipexole, 8 ropinirole, 1 rotigotine), and other antiparkinsonian medications (*n* = 8; 7 amantadine, 1 rasagiline, 1 selegiline). All patients were tested in their “on” state. Levodopa equivalent dose (LED) was calculated by the formula of Tomlinson *et al.* (34). Demographic and clinical features of participants are given in Table 1.

**Table 1:** Demographic and Clinical Features and Neuropsychological Measures (Means, Standard Deviations, and Group Differences)

Features/Measures	Parkinson’s Disease	Control Subjects	Group Difference
Male:Female	11:10	12:8	ns
Age (Years)	64.0 (8.1)	65.3 (5.7)	ns
Education (Years)	14.6 (3.8)	15.1 (2.5)	ns
Mini Mental State Examination	28.9 (1.2)	29.3 (.9)	ns
Duration of Symptoms (Years)	10.8 (4.9)	–	–
UPDRS I: Mentation, Behavior and Mood	8.8 (4.9)	–	–
UPDRS III: Motor	20.6 (7.7)	–	–
Hoehn and Yahr Scale	1.9 (.4)	–	–
Schwab and England Activities of Daily Living Scale	86.5 (5.9)	–	–
Levodopa Actual Dose (mg/day)	393.4 (221.0)	–	–
Levodopa Equivalent Dose (mg/day)	632.6 (310.6)	–	–
Beck Depression Inventory II	9.9 (5.5)	3.8 (3.9)	*p* < .001
Epworth Sleep Scale	9.3 (5.3)	5.1 (2.9)	*p* < .01
Insomnia Severity Index	9.2 (5.8)	3.1 (2.8)	*p* < .001
REM Sleep Behaviour Disorder Screening Questionnaire	6.4 (2.9)	2.0 (1.5)	*p* < .001
Spot-the-Word Test	112.8 (13.6)	121.2 (10.3)	*p* < .05
Category Fluency Test	20.6 (4.9)	24.3 (6.7)	*p* < .05
Letter Fluency Test	17.0 (5.6)	18.1 (4.5)	ns
Forward Digit Span	7.0 (1.1)	7.2 (.8)	ns
Backward Digit Span	5.4 (1.5)	5.7 (1.3)	ns
Simple Reaction Time (msec)	293.7 (53.0)	314.4 (71.8)	ns
Choice Reaction Time (msec)	353.4 (47.3)	392.2 (70.0)	*p* < .05

Group difference: *p* values of chi-squared or two-sample *t* tests as appropriate (two-tailed; ns, *p* > .1).

ns, not significant; UPDRS, Unified Parkinson’s Disease Rating Scale.

Twenty healthy control subjects with no history of significant neurologic or psychiatric disorder were recruited from the Cambridge University PD Research Clinic database and the Cognition and Brain Sciences Unit volunteer panel. This study was approved by the local research ethics committee and exempted from clinical trials status by the Medicines and Healthcare Products Regulatory Agency. Written informed consent was obtained from all participants.

### Experimental Design

A double-blinded randomized crossover design was used, with separate sessions at least 6 days apart, including a neuropsychological battery and brain imaging, after either 40 mg oral atomoxetine or an identically overcoated placebo capsule. In humans, plasma concentration of atomoxetine peaks approximately 2 hours after a single oral dose (35). Blood samples, therefore, were collected 2 hours after administration, immediately before fMRI scanning in each session (plasma concentration after atomoxetine: mean 444 ng/mL, range 32–889 ng/mL; under placebo: 0 ng/mL). Control subjects underwent one testing session only without drug or placebo.

The inhibition task included randomly interleaved action restraint and cancellation trials, which were matched for drug levels, practice effects, and fatigue. There were 360 Go trials (75%), 40 NoGo trials (8%), and 80 stop-signal trials (SS) (17%). In Go trials, participants responded to a left/right black arrow (duration 1000 msec) by pressing left/right buttons with their right hand. In SS trials, the left/right black arrow changed color (from black to red) concurrent with a tone, after a short variable stop-signal delay, indicating the need to cancel the response. The stop-signal delay was varied from trial to trial in steps of 50 milliseconds by a tracking algorithm to maintain 50% successful inhibition (15). In NoGo trials, participants were required to make no response to a red left/right arrow (duration 1000 msec) and concurrent tone, equivalent to a stop-signal delay of zero. Preliminary studies in 20 healthy adults indicated that performances and activations were preserved in the combined task compared with separate NoGo and stop-signal tasks.

For this task, four key parameters of interest were measured: the rate of Go commission errors, mean reaction time of correct Go trials (Go RT), rate of NoGo commission errors, and SSRT. For Go trials, a commission error occurred when participants mistakenly pressed the opposite buttons. For NoGo trials, a commission error means participants pressed a button. Both error rates were arcsine transformed for further analysis. The SSRT was estimated by subtracting mean stop-signal delay from finishing time of the stop process (using the integration method) (36). The finishing time is the *n*th Go RT, where Go RTs are rank-ordered and *n* is determined by the probability of responding, *p(respond|signal)*, and the number of correct Go trials, *m*, as *n = m × p(respond|signal)*. We also monitored omission errors on Go trials. Patients showed higher rates of Go omission errors than control subjects (placebo 7%, atomoxetine 9%, control 4%, *p* < .01). Analysis of the Go RT distribution indicated that omission errors were not due to excessive response times but other impairments (e.g., slipping off the buttons or failure to press the button). Although the omissions can only be detected for Go trials, their occurrence in SS trials may affect SSRT estimation, as *p(respond|signal)* would be underestimated. The tracker algorithm may also elevate the stop-signal delay. We corrected the observed *p(respond|signal)* by individual subject’s Go omission rate using equation 1 below (written communication, G. Logan, Ph.D., 2013). Correction of NoGo error rate used equation 2.

(1) Correctedp(respond|signal)=Observedp(respond|signal)/(1−Goomissionrate)

(2) CorrectedNoGoerrorrate=ObservedNoGoerrorrate/(1−Goomissionrate)

Disease effects on behavioral indices were examined by two-sample *t* tests (PD-placebo > control). To investigate drug effects on SSRT and NoGo error rate, repeat-measures analyses of variance were conducted with drug (atomoxetine vs. placebo) as a within-subject factor and disease severity (Unified Parkinson’s Disease Rating Scale [UPDRS] motor), age, LED, and plasma concentration as covariates. Note that the disease effect refers to treated Parkinson’s disease (37, 38). In principle, differences between PD-placebo and control subjects could be due to the presence of PD, the use of medication, or in our design, the additional use of a placebo tablet in the PD-placebo group. However, the concurrent use of dopaminergic drugs is unlikely to fully explain the group differences (30).

Participants also completed the Beck Depression Inventory II, Epworth Sleepiness Scale, Insomnia Severity Index, REM Sleep Behaviour Disorder questionnaire, Spot-the-Word test, category and letter fluency tests, forward and backward digit spans, and simple and choice reaction times.

### fMRI Data Acquisition and Analysis

Magnetic resonance imaging used a Siemens Trio 3T scanner (Siemens, München, Germany). Functional images were acquired using a quiet echo planar imaging sequence (2656-msec repetition time, 44-msec echo time, 78° flip angle, 32 sequential descending oblique axial slices, 192 × 192 mm^2^ field of view, 3-mm thickness, .75-mm gap, and 3 × 3 mm^2^ in-plane resolution) (39). High-resolution T1-weighted magnetization-prepared rapid-acquired gradient echo images were acquired (144 sequential sagittal slices, 240 × 240 mm^2^ field of view, 1.25-mm thickness, and 1.25 × 1.25 mm^2^ in-plane resolution).

Functional MRI analysis used SPM8 (Functional Imaging Laboratory, London, United Kingdom; www.fil.ion.ucl.ac.uk/spm). Eleven volumes were discarded to allow magnetization equilibration. Functional images were realigned to the first image, sinc-interpolated to correct for differences in slice acquisition time, and normalized to the Montreal Neurological Institute (MNI) template using iterative segmentation. Normalized images were spatially denoised using a wavelet-based three-dimensional denoising approach, which not only suppresses noise but also preserves essential spatial details (edge and shape of activation) that are often suppressed in Gaussian smoothing (40).

A univariate analysis examined the effects of disease and drug on activation. Subject-level general linear models convolved a design matrix with the canonical hemodynamic response function. Event types included in the design matrix were correct SS, NoGo, and Go trials and commission and omission error trials. Six movement parameters were included as nuisance regressors. Classical parameter estimation was applied with a one-lag autoregressive model and high-pass filter of 128 seconds. Contrasts of interest, SS > Go and NoGo > Go, were entered into one-sample *t* tests for stop-related and NoGo-related activations of each group (voxel-level *p* < .001 uncorrected, cluster-level *p* < .05 familywise error [FWE]-corrected).

In view of our anatomically constrained hypotheses regarding disease and drug effects, a study-specific RIFG region of interest (ROI) was built. This ROI is the intersection of the anatomical RIFG (Automated Anatomical Labeling based [Automated Anatomical Labeling, AAL, Caen University, France]) and the stop-related (SS > Go) or NoGo-related (NoGo > Go) activations in control subjects. Disease effects on stop-related and NoGo-related RIFG activations were examined by two-sample *t* tests with small volume correction (PD-placebo < control, voxel-level *p* < .05 FWE-corrected). To investigate drug effects, betas (parameter estimates) of the RIFG ROI were pooled in repeated-measures analyses of variance with drug as a within-subject factor and UPDRS motor, age, LED, and plasma concentration as covariates.

A functional connectivity analysis examined whether the interaction between the RIFG and striatum was modulated by the disease and/or atomoxetine. Physiological fMRI signals were extracted from a spherical RIFG ROI (unbiased ROI with 6-mm radius, centered at the RIFG peak of SS > Go in all subjects, i.e., a one-sample *t* test combining control subjects and patients) and included in another subject-level general linear model (physiological regressor). Estimated betas indicated the degree to which the time course of a voxel correlated with the RIFG time course. One-sample *t* tests showed regions positively correlated with the RIFG in each group (voxel-level *p* < .001 uncorrected, cluster-level *p* < .05 FWE-corrected). The effects of disease (PD-placebo < control) and drug (PD-atomoxetine > placebo) were examined by two-sample and paired-sample *t* tests, respectively (small volume correction, voxel-level *p* < .05 FWE-corrected).

### Diffusion MRI

Diffusion-weighted images were collected along 63 gradient directions (single acquisition, 63 sequential interleaved ascending axial slices, 192 × 192 mm^2^ field of view, 2-mm thickness, and 2 × 2 mm^2^ in-plane resolution) and analyzed with FSL4.1 (FMRIB software library, FMRIB, Oxford, United Kingdom; www.fmrib.ox.ac.uk/fsl). Images were corrected for head movements and eddy currents and smoothed with a 2.5-mm Gaussian kernel. Diffusion tensors were linearly fitted to the diffusion-weighted images, and fractional anisotropy (FA) images were calculated for tract-based spatial statistics (41). The FA images were corrected for outlier values, registered to the FMRIB58_FA template, and normalized to the MNI 152 space. A mean FA skeleton was derived and thresholded at FA > .2 to represent the center of the white matter tracts common to all subjects. Permutation tests with threshold-free cluster enhancement (42) examined whether the effects of atomoxetine on SSRT varied with the strength of frontostriatal connections. Based on our hypothesis of frontostriatal interactions between inhibition and motor systems, we focused on the right anterior internal capsule, which contains fibers connecting the frontal lobe, striatum, and thalamus (ROI based on the Johns Hopkins University diffusion tensor imaging-based white-matter atlas).

## Results

### Behavioral Results

Results of the neuropsychological battery are given in Table 1. Groups were well matched by age, sex, education, and Mini Mental State Examination. As is typical of PD, sleep and depression symptom scores were higher and category (semantic) fluency was reduced in patients (33).

Table 2 shows the SSRT, Go RT, Go error rate, and NoGo error rate in each group. Compared with control subjects, PD-placebo had longer SSRT and more NoGo errors and Go errors. The group difference in SSRT could not be attributed to a difference in Go RT. Although there was no groupwise drug effect, the behavioral benefit of atomoxetine was conditional on individual differences in brain activation and functional and anatomical connectivity (see below).

**Table 2:** Performance on the Stop-Signal and NoGo Tasks

Parameters	Control Subjects	PD-PLA	PD-ATO	Disease Effect	ATO Effect (Whole Group)
SSRT (msec)	142 (44)	167 (50)	181 (85)	*p* < .05	ns
Go RT (msec)	532 (129)	554 (108)	562 (104)	ns	ns
NoGo Error (rad)	.06 (.13)	.14 (.13)	.13 (.15)	*p* < .05	ns
Go Error (rad)	.08 (.05)	.14 (.06)	.15 (.07)	*p* < .001	ns

Values are group means (standard deviations). Disease effect refers to the contrast PD-PLA vs. control subjects (two-sample *t* tests), which differs by the presence of Parkinson’s disease and concurrent dopaminergic/placebo medication in patients. ATO effect refers to the contrast PD-ATO vs. PD-PLA (repeated-measures analyses of variance with age, Unified Parkinson’s Disease Rating Scale, levodopa equivalent dose, and plasma concentration as covariates). ns, *p* > .1.

ATO, atomoxetine; ns, not significant; PD, Parkinson’s disease; PLA, placebo; rad, arcsin transformed to radians; RT, reaction time; SSRT, stop-signal reaction time.

### fMRI Results

**Figure 1:**
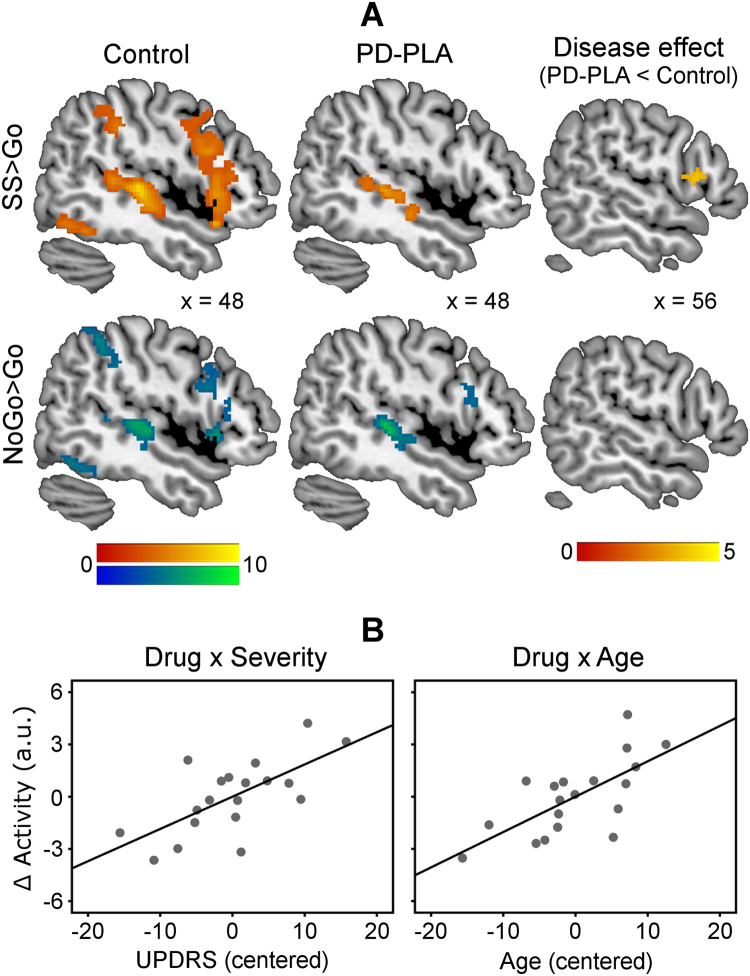
**(A)** Control subjects showed greater stop-related (stop-signal [SS] > Go, warm colors) and NoGo-related activations (NoGo > Go, cool colors) in the right inferior frontal gyrus (RIFG). The stop-related RIFG activation was significantly weaker in Parkinson’s disease-placebo (PD-PLA) than in control subjects (disease effect, *p* < .05 small-volume corrected). Color scales indicate *t* values. Coordinates are in Montreal Neurological Institute space. **(B)** Atomoxetine selectively enhanced the stop-related RIFG activation in more advanced disease (drug × severity) and in older patients (drug × age). The atomoxetine-induced change of RIFG activity (∆Activity) was positively correlated with Unified Parkinson’s Disease Rating Scale (UPDRS) and age (mean-corrected data).

Figure 1 presents inhibition-related activations, including the RIFG, in each group. Control subjects showed RIFG activations for SS > Go (peak coordinates in MNI space [54 18 8], *t* = 7.17, 1621 voxels) and for NoGo > Go (peak [48 18 −2], *t* = 6.00, 452 voxels; Figure 1A). The stop-related RIFG activation (SS > Go), not the NoGo-related activation (NoGo > Go), was significantly reduced in PD-placebo compared with control subjects (disease effect, peak [56 16 12], *t* = 3.88, 14 voxels; Figure 1A).

The atomoxetine effect on stop-related RIFG activation showed an interaction between drug and UPDRS motor (*F* = 11.78, *p* < .005), as well as an interaction between drug and age (*F* = 12.56, *p* < .005). This indicates that atomoxetine enhanced RIFG activations in more advanced disease and in older patients (Figure 1B). There was no effect of LED or plasma concentration in the multiple regression model (see also Figure S2 in Supplement 1). No drug effect was obtained on NoGo-related RIFG activation.

The NoGo and SSRT tasks are associated with widespread activations beyond the RIFG (43, 44). The RIFG is the focus of this study, but we also analyzed the drug effects on other areas including the left inferior frontal gyrus, supplementary motor area, striatum, and thalamus. The results are presented in the details in Supplement 1. In brief, we observed an effect of atomoxetine in none of these areas except the thalamus (Figure S1 in Supplement 1).

**Figure 2:**
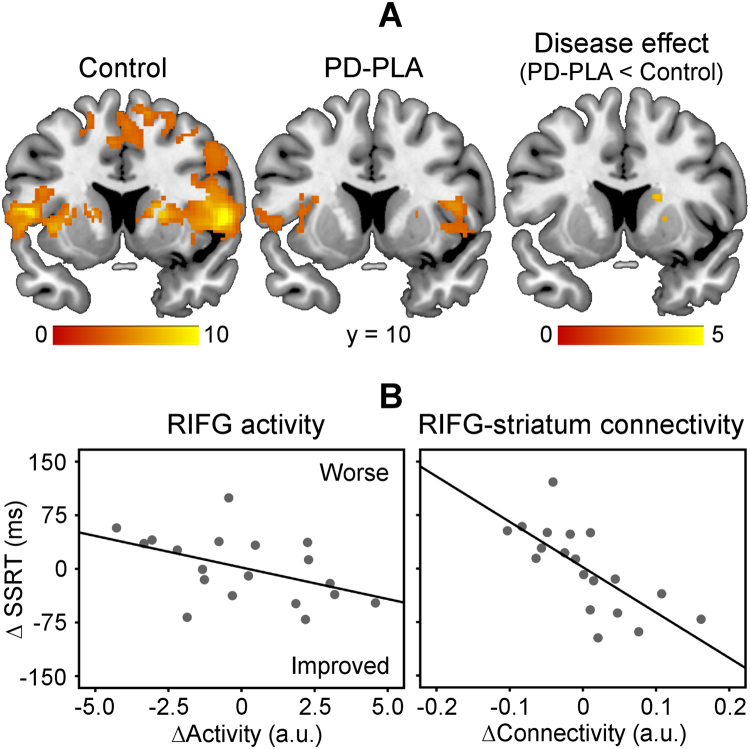
**(A)** Control subjects showed strong functional coupling between the right inferior frontal gyrus (RIFG) and striatum. The RIFG-striatum connectivity was significantly weaker in Parkinson’s disease-placebo (PD-PLA) than in control subjects (disease effect, *p* < .05 small-volume corrected). Color scales indicate *t* values. Coordinates are in Montreal Neurological Institute space. **(B)** The atomoxetine-induced change of stop-signal reaction time (SSRT) (∆SSRT) was related not only to the change of RIFG activity (∆Activity, *p* < .05) but also to the change of RIFG-striatum connectivity (∆Connectivity, mean-corrected data, *p* < .005).

Figure 2A presents areas functionally correlated positively with the RIFG, including the bilateral striatum. Control subjects showed strong coupling between the RIFG and right striatum (peak [20 8 8], *t* = 9.16, 537 voxels). The interregional interaction was significantly reduced in PD-placebo compared with control subjects (disease effect, peak [18 10 16], *t* = 3.87, 13 voxels). Atomoxetine did not restore the frontostriatal connectivity at the group level.

To determine whether change of behavioral performance was related to atomoxetine’s enhancement of RIFG activity and/or RIFG-striatum connectivity, a linear regression model (equation 3) used the change of SSRT as a dependent variable (∆SSRT: SSRT-atomoxetine vs. SSRT-placebo). Independent variables were the change of RIFG activity in stop-signal trials (∆Activity, activity betas from the study-specific RIFG ROI), the change of RIFG-striatum connectivity (∆Connectivity, connectivity betas from the significant cluster of right striatum in control subjects), and the baseline SSRT under placebo (SSRT-placebo).

(3) $$\;\Delta \mathrm{SSRT}=\beta_{1}\times \Delta \mathrm{Activity}+\beta_{2}\times \Delta \mathrm{Connectivity}+\beta_{3}\;\times \mathrm{SSRT}-\mathrm{placebo}$$

The model was significant overall (*F* = 8.97, *p* = .001). Figure 2B illustrates that ∆SSRT was related to both ∆Activity (β_1_ = −.37, *t* = −2.14, *p* < .05) and ∆Connectivity (β_2_ = −.63, *t* = −4.11, *p* = .001), in addition to the SSRT-placebo (β_3_ = .61, *t* = 3.49, *p* < .005). It suggests that the behavioral effect of atomoxetine depends not only on changes in frontal activity but also on changes in frontostriatal connectivity.

**Figure 3:**
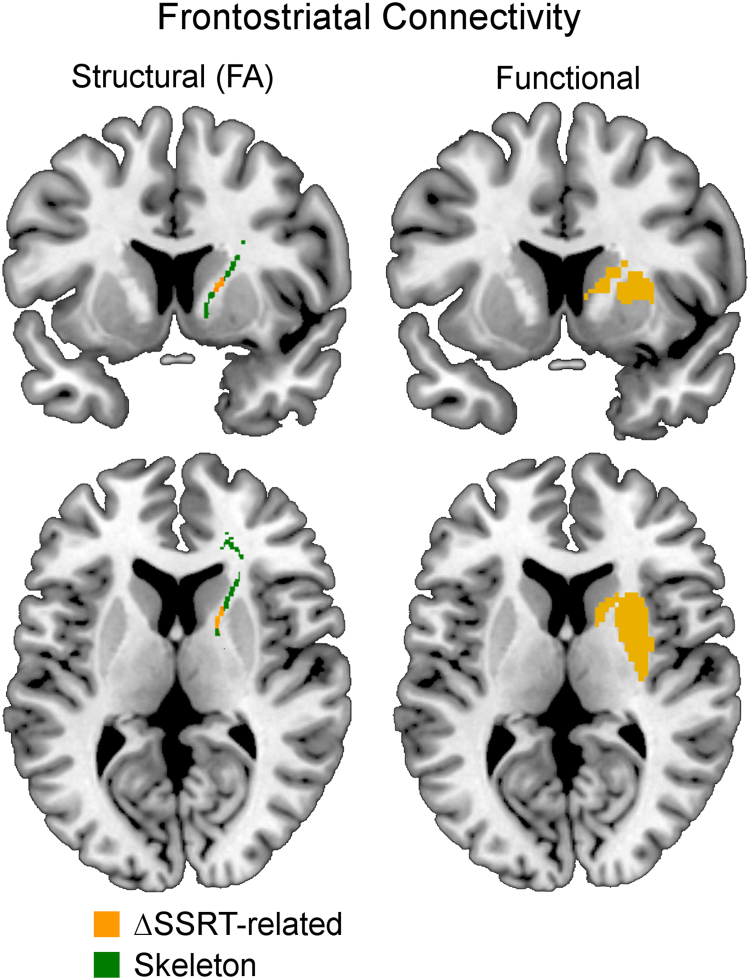
The behavioral benefit of atomoxetine (reduction in stop-signal reaction time, ΔSSRT) was related not only to the functional connectivity but also to the structural connectivity between the frontal cortex and striatum (fractional anisotropy [FA] of white matter, *p* < .05 threshold-free cluster enhancement-corrected within anterior-internal capsule skeleton).

In addition to changes of functional connectivity, the strength of structural connectivity between the frontal cortex and striatum predicted atomoxetine’s effect on performance (Figure 3). The SSRT change (∆SSRT) was negatively correlated with the fractional anisotropy of white matter in the anterior limb of the internal capsule: a reduction in SSRT was observed in patients with greater anatomical frontostriatal connectivity.

## Discussion

This study investigated effects of the selective noradrenaline reuptake inhibitor atomoxetine on response inhibition in patients with PD. We confirmed that patients with PD and on dopaminergic medication were impaired at response inhibition, even in the absence of impulse control disorders, with longer SSRT and more NoGo errors than healthy control subjects. Behavioral impairments were associated with reduced RIFG activations on successful stop-signal trials and weaker functional connectivity between the RIFG and striatum. The group differences might arise from the presence of neuropathology or concurrent optimized motor therapy by dopaminergic drugs. Atomoxetine modulated the stop-related RIFG activation in PD, similar to its enhancement of RIFG activation in another impulsive disorder, attention-deficit/hyperactivity disorder (ADHD) (11). Enhanced activation emerged in patients with more severe disease. Moreover, improvements in stop-signal performance correlated not only with atomoxetine’s effect on frontal activation but also with its effect on frontostriatal connectivity.

These observations build on studies of atomoxetine in healthy adults and those with ADHD (15, 19), confirming that noradrenaline plays an important role in regulating response inhibition. However, we propose that enhancement of prefrontal activation is not sufficient to improve behavioral inhibition, unless frontostriatal functional connectivity is also enhanced. This would explain why the behavioral benefits of atomoxetine were marked in healthy adults (19) with intact corticostriatal connectivity but not at the group level with PD or ADHD (11), where frontostriatal connectivity is often impaired (7). Moreover, patients vary in their responses to noradrenergic or other treatments. The effectiveness of treatment may be predicted if impairments of regional function and network communication are considered jointly, including anatomical frontostriatal connectivity. Functional and/or structural connectivity might therefore be used in the stratification of patients for treatment and in prediction of efficacy.

It might at first seem paradoxical that atomoxetine enhanced stop-related RIFG activation in patients with relatively more advanced disease, while reducing SSRT in patients with stronger pretreatment frontostriatal structural connectivity. However, these results are consistent. First, responses to atomoxetine may be strongly influenced by the baseline noradrenergic state. A Yerkes-Dodson model (of an inverted U-shaped function) has been proposed to account for the nonlinear relation between noradrenaline levels and task performances in animal studies (45, 46). In this model, the performance is optimal with intermediate noradrenaline levels but impaired at excess noradrenaline levels. In a previous study of an independent PD cohort, we observed this pattern of responses to atomoxetine in PD: low plasma concentration improved response inhibition, whereas high concentration impaired response inhibition (29). We propose that our dose of atomoxetine represents a replacement therapy for patients who have lost a significant proportion of noradrenergic capacity (5). But for patients with mild disease and minor changes in intrinsic noradrenaline transmission, the same atomoxetine dose represents in effect an overdose. Second, for atomoxetine to be able to exert a behavioral benefit, the RIFG must be effectively connected with the motor systems that ultimately generate behavioral outputs. Because the striatum modulates the execution of actions in response to the cortical motor systems (47), the relative preservation of frontostriatal connectivity improves the ability to relay inhibition commands. It is worth noting that neither UPDRS nor frontostriatal connectivity represents a gold standard biomarker for the stage of disease. While both would be expected to change when disease progresses, they might not be driven by the same biological factors. For example, UPDRS might be higher in some patients because of signs that are not directly related to the frontostriatal connectivity (e.g., tremor or balance). In our cohort, UPDRS motor scores and diffusion tensor imaging measures of frontostriatal connectivity were not significantly correlated.

Atomoxetine did not significantly affect NoGo activation or behavior. While this could be due to type II error, it is also consistent with preclinical studies (20, 21) reporting stop-signal modulation by noradrenaline (15, 19) and NoGo modulation by serotonin (48). This neuropsychopharmacologic specificity provides a reassuring internal control for the observed effects of atomoxetine.

The role of noradrenaline extends beyond response inhibition (8). Previous studies have linked the noradrenergic system with other executive functions, such as cognitive flexibility in response to a changing environment (10). For example, attentional set-shifting in the Wisconsin Card Sorting Test and Cambridge Neuropsychological Test Automated Battery intradimensional/extradimensional set-shifting task are impaired by PD (49, 50) and in animals with selective depletion of cortical noradrenaline (51, 52) but can be relieved by atomoxetine (53, 54). Noradrenergic systems may therefore support multiple executive processes, including control of motor and nonmotor impulsivity, response inhibition, and cognitive flexibility (21, 55), which requires further evaluation for PD.

There are clinical, methodological, and pharmacologic limitations with this study. First, our investigation was limited to response inhibition rather than other dimensions of impulsivity, such as reflection impulsivity or impairment of decisions based on risk and rewards. The latter have been strongly associated with dopaminergic dysfunction in treated PD (1, 2, 3). Second, we relied on clinical diagnostic criteria because the United Kingdom PD Society Brain Bank Criteria have high sensitivity and specificity. Clinically, none of our patients had impulse control disorders (ICDs). But, we note that PD causes impulsivity and response inhibition deficits even without ICDs (30, 31, 56). Further studies are needed to test whether atomoxetine can improve impulsivity in patients with ICDs. Third, fMRI measures a hemodynamic response to neural activity and in principle atomoxetine might alter fMRI signals through affecting regional cerebral blood flow. However, the frontal regions and striatum retain normal blood flow under atomoxetine (57). Moreover, the cognitive and anatomical specificity of the effects we observed make a significant direct or generic effect of atomoxetine on the neurovascular response unlikely.

Finally, we must consider the neuropharmacologic complexity of patients and atomoxetine. Our data alone cannot determine whether the effects of atomoxetine are via cortical reuptake inhibition (most likely) (23, 55), from changes in phasic-to-tonic firing ratios in the locus coeruleus (45), or through antagonism of glutamate receptors (58). We recognize that atomoxetine can increase extracellular dopamine levels (59) and our patients took levodopa and/or dopamine agonists. But the atomoxetine effect is unlikely mediated by indirect dopaminergic mechanisms. First, as we have shown with multiple regression models, the atomoxetine effect on activation was independent of LED. Second, the specific effect of dopamine on stop-signal inhibition is unclear. Some studies of SSRT have used methylphenidate, which increases extracellular levels of both dopamine and noradrenaline, leaving it ambiguous as to whether dopamine or noradrenaline is responsible for the beneficial effect (11, 24, 60, 61). Studies that used more selective dopaminergic agents, such as levodopa and dopamine transporter blocker, have observed minor effects on SSRT, and patients with PD have poor response inhibition both on and off medication (12, 30, 55, 62). The advent of more selective noradrenergic drugs will further address these issues in the future.

In conclusion, the noradrenergic reuptake inhibitor atomoxetine can improve response inhibition in a subgroup of patients, especially when it is associated with enhanced stop-signal activation in the inferior frontal cortex (which is more likely with relatively more advanced disease). The behavioral measure or response inhibition efficacy was improved (i.e., shorter SSRT) for patients in whom frontostriatal functional connectivity was also enhanced by atomoxetine or in those with relatively preserved frontostriatal structural connectivity. These findings contribute to the broader understanding of the importance of noradrenergic systems for executive functions and point the way to new stratified clinical trials of noradrenergic therapies in selected patients with Parkinson’s disease.
