# Supplementary material for: Improving Response Inhibition in Parkinson’s Disease with Atomoxetine
Source: Biol Psychiatry. 2015 Apr 15;77(8):740–8. doi: 10.1016/j.biopsych.2014.01.024 (PMC4384955; doi:10.1016/j.biopsych.2014.01.024)
Supplement: Supplementary file 1 — Supplementary Material [file mmc1.pdf]

Improving Response Inhibition in Parkinson's Disease with Atomoxetine

Supplemental Information

We compared patients who received placebo on their first visit (Placebo-1st) and patients who received placebo on their second visit (Placebo-2nd, Table S1). There was no significant difference between the two Parkinson's disease (PD) sub-groups, or between controls and Placebo-1st (chi-squared or two-sample *t*-tests as appropriate, $p < 0.05$ uncorrected for multiple comparison). Stop-Signal Reaction Time (SSRT) and NoGo error rates were larger in Placebo-2nd than in controls. However, the direction of effect suggests that the difference is not driven by practice, since the absolute reaction times and error rates were in the direction of worse performance.

Table S1. Comparisons between patients who received placebo on their first visit and patients who received placebo on their second visit (means, standard deviations and group differences)

Features / Measures	Placebo-1st	Placebo-2nd	Controls
Male:Female	5:6	6:4	12:8
Age (years)	64.4 (8.5)	63.7 (8.2)	65.3 (5.7)
UPDRS III motor	20.5 (7.1)	20.5 (9.1)	-
Levodopa equivalent dose (mg/day)	565.4 (210.0)	748.5 (382.0)	-
SSRT (ms)	154 (46)	181 (53)	142 (44)
Go RT (ms)	523 (74)	592 (134)	532 (129)
NoGo error (rad)	0.09 (0.13)	0.20 (0.12)	0.06 (0.13)

RT, reaction time; SSRT, Stop-Signal Reaction Time; UPDRS, Unified Parkinson's Disease Rating Scale.

Table S2. Peaks of brain activations for “successful Stop-Signal > Go”

Regions	Hemisphere	MNI coordinates x	y	z	T	# of voxels
Controls						
Inferior frontal gyrus & insula	L	-36	20	-12	10.53	756
	R	46	18	-16	8.52	1621
Supplementary motor area	R	8	22	38	5.30	347
Superior temporal gyrus	L	-46	-24	-2	6.98	261
	R	56	-30	4	11.53	1718
Middle temporal gyrus	L	-60	-44	8	5.73	337
Angular gyrus, supramaginal gyrus & inferior parietal lobule	L	-30	-58	48	4.69	134
	R	48	-36	38	5.66	488
Fusiform area & inferior occipital gyrus	L	-34	-88	-4	6.57	892
	R	38	-64	-16	6.82	426
PD under placebo						
Insula	L	-32	20	-4	5.49	96
	R	34	24	-4	5.82	110
Supplementary motor area	R	8	14	42	6.14	83
Superior temporal gyrus	L	-52	-10	-10	9.59	681
	R	52	-28	2	6.92	886
Inferior parietal lobule	R	40	-52	40	5.12	90

Whole-brain results are shown at voxel-level $p < 0.001$ uncorrected and cluster-level $p < 0.05$ family-wise-error-corrected.

MNI, Montreal Neurological Institute; PD, Parkinson’s disease; L, left; R, right.

Table S3. Peaks of regional brain activations for “NoGo > Go”

Regions	Hemisphere	MNI coordinates			T	# of voxels
		x	y	z		
Controls						
Inferior frontal gyrus	L	-40	6	26	6.66	93
	R	44	14	22	6.06	452
Superior temporal gyrus	L	-60	-42	8	7.43	560
	R	62	-32	12	7.72	1427
Superamarginal gyrus & inferior parietal lobule	L	-28	-66	38	4.90	129
	R	60	-42	40	6.41	578
Fusiform area & inferior occipital gyrus	L	-34	-88	-10	8.22	848
	R	34	-88	-6	7.41	1001
PD under placebo						
Inferior frontal gyrus	R	45	20	18	5.54	110
Superior temporal gyrus	L	-48	-30	2	7.71	875
	R	64	-28	6	9.70	1311
Inferior occipital gyrus	L	-20	-102	-6	6.41	519
	L	22	-94	0	6.66	492

Whole-brain results are shown at voxel-level $p < 0.001$ uncorrected and cluster-level $p < 0.05$ family-wise-error -corrected.
See Table S2 for abbreviations.

Table S4. Group difference in brain activity for “successful Stop-Signal > Go”

Regions	Hemisphere	MNI coordinates			T	# of voxels
		x	y	z		
Control > PD-placebo for stop-related activity						
Inferior frontal gyrus	R	56	16	12	3.88	31
Middle occipital gyrus	L	-24	-92	14	4.81	44
	R	28	-94	16	4.69	60

Whole-brain results are shown at voxel-level $p < 0.001$ uncorrected and cluster-level $p < 0.05$ uncorrected. Under this threshold, there was no group difference for NoGo-related (NoGo > Go) activity.
See Table S2 for abbreviations.

Table S5. Brain activations for "unsuccessful Stop-Signal > Go"

Region	Hemisphere	MNI coordinates			T	# of voxels
		x	y	z		
Controls						
Fusiform area & inferior occipital gyrus	L	-24	-96	-2	7.94	941
	R	40	-58	-16	6.75	310

Whole-brain results were showed at voxel-level $p < 0.001$ uncorrected and cluster-level $p < 0.05$ family-wise-error -corrected. No activation was observed in PD under this threshold.
See Table S2 for abbreviations.

Additional Region of Interest (ROI) Analysis

Effects of Atomoxetine in Other Brain Areas

The NoGo and SSRT tasks were associated with widespread activations beyond the right inferior frontal gyrus (RIFG), in our study and previous reports. We therefore investigated *post hoc* the effects of atomoxetine on other brain areas including the left inferior frontal gyrus (LIFG), supplementary motor area (SMA), striatum and thalamus.

The LIFG and SMA ROIs were defined as the intersection of the AAL-based anatomical LIFG or SMA and the SS > Go activation in controls (one-sample *t*-test, voxel-level $p < 0.001$ uncorrected, cluster-level $p < 0.05$ FWE-corrected). These ROIs are therefore independent of the patient data with which we sought an effect of drug.

The striatum and thalamus ROIs were anatomically defined because neither region was observed for SS > Go in controls at our standard threshold in this study (Table S2). Parameter estimates (beta values in SPM) of SS > Go in PD were entered into repeated-measures ANOVAs with drug as a within-subject factor and Unified Parkinson's Disease Rating Scale (UPDRS) motor, age, levodopa equivalent dose and plasma concentration as covariates (similar to the RIFG ROI analysis in the main text).

There was no effect of atomoxetine in the LIFG, SMA or striatum. However, the thalamus showed a drug-by-UPDRS interaction ($F = 5.79$, $p < 0.05$) and a drug-by-age interaction ($F = 12.12$, $p < 0.005$; Figure S1). This indicates that atomoxetine modulated the stop-related thalamic activation in advanced disease and in older patients.

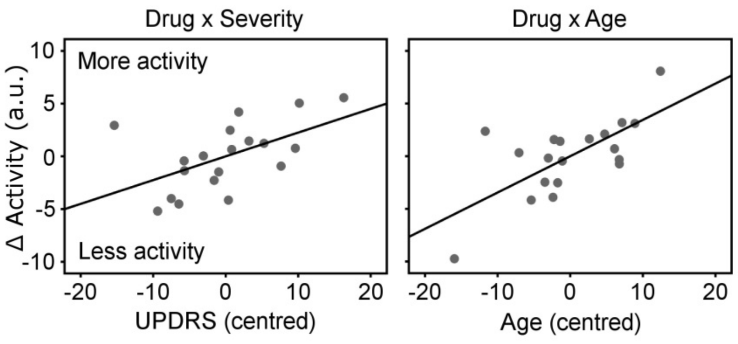

Figure S1. Effects of atomoxetine in the thalamus.

Effect of Atomoxetine Concentration

The plasma concentration is a factor that could influence the impact of atomoxetine on behavior and brain activity. For example, Chamberlain *et al.* (1) observed a positive correlation between the plasma concentration of atomoxetine and RIFG activation in healthy adults.

In the main analysis, we used multiple regression models to examine the factors that influence performance and activation. In the presence of multiple variables, which partially correlate, the multiple regression approach is preferred over a set of simple correlations. Using these multiple regression models, we identified the UPDRS motor, age and frontostriatal connectivity, but not the plasma concentration, as explanatory variables for regional frontal activation (main text page 11, Figure 1).

However, in view of Chamberlain *et al.* (1), we also investigated the effect of atomoxetine concentration on RIFG activation, in a simple regression model (correlating activation with atomoxetine concentration in PD). We extracted parameter estimates (betas) from the RIFG peak of activation for Stop-Signal trials (vs. baseline; cf. (1)) from patients, during their atomoxetine session (peak: [46 14 22]; black circle in Figure S2; voxel-level $p < 0.001$ uncorrected and cluster-level $p < 0.05$ FWE-corrected).

The result shows that, like Chamberlain *et al.* (1), there was a positive correlation between the plasma concentration of atomoxetine and RIFG activation ($r = 0.43$, $p < 0.05$). One patient achieved a very low plasma concentration after the standard oral dose of atomoxetine (32 ng/ml, far left point). If this individual is excluded *post hoc*, the correlation is 0.62 ($p < 0.005$).

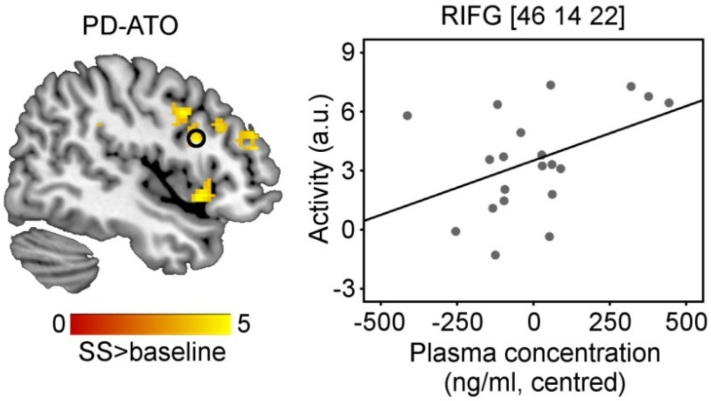

Figure S2. Effect of atomoxetine (ATO) level on right inferior frontal gyrus (RIFG) activation. PD, Parkinson's disease; SS, stop-signal trials.

Supplemental Reference

1. Chamberlain SR, Hampshire A, Müller U, Rubia K, Del Campo N, Craig K, *et al.* (2009): Atomoxetine modulates right inferior frontal activation during inhibitory control: a pharmacological functional magnetic resonance imaging study. *Biol Psychiatry*. 65:550-555.
